# Evaluating the effects of pharmaceutical pollutants on common carp (*Cyprinus carpio*): histopathological and antioxidant responses

**DOI:** 10.3389/fphys.2025.1557647

**Published:** 2025-04-29

**Authors:** Walaa M. Shaalan, Shaimaa K. A. Idriss, Jae-Seong Lee, Nadia H. Mohamed, Alaa El-Din H. Sayed

**Affiliations:** ^1^ Zoology Department, Faculty of Science, Benha University, Benha, Egypt; ^2^ Bioinformatics Group, Faculty for Biology and Biotechnology and Center for Protein Diagnostics, Ruhr-University, Bochum, Germany; ^3^ Department of Aquatic Animal Medicine and Management, Faculty of Veterinary Medicine, Assiut University, Assiut, Egypt; ^4^ Department of Biological Sciences, College of Science, Sungkyunkwan University, Suwon, Republic of Korea; ^5^ Department of Biology, Faculty of Science, Jazan University, Jazan, Saudi Arabia; ^6^ Department of Zoology, Faculty of Science, Assiut University, Assiut, Egypt; ^7^ Molecular Biology Research and Studies Institute, Assiut University, Assiut, Egypt

**Keywords:** pharmaceutical pollutants, aquatic ecosystems, *Cyprinus carpio*, neurological effects, immunological response

## Abstract

**Introduction:**

The release of pharmaceutical chemicals into aquatic environments has emerged as a significant ecological concern, originating from agricultural runoff, sewage effluents, and improper disposal of medications.

**Methods:**

This study investigates the impacts of four common pharmaceuticals (bromazepam, naproxen, metoprolol, and sotalol) on common carp (*Cyprinus carpio*), a vital bioindicator species. We evaluated neurological, immunological, and histopathological responses in carp exposed to these pharmaceuticals over 15 days.

**Results:**

Neurological assessments showed significant reductions in acetylcholinesterase (AChE) and monoamine oxidase (MAO) activities, and nitric oxide (NO) levels, indicating potential disruptions in neurotransmission and enzyme function. Immunological analysis revealed elevated levels of pro-inflammatory cytokines interleukin-1β (IL-1β) and interleukin-6 (IL-6), suggesting an inflammatory response. Histopathological examinations identified tissue alterations in the liver, kidney which correlated with the observed biochemical and immune responses.

**Discussion:**

These findings highlight the adverse effects of pharmaceutical contaminants on aquatic species, emphasizing the necessity for comprehensive environmental risk assessments and strategies to mitigate their impact. This study enhances the understanding of pharmaceutical pollutants’ ecological effects, informing policy and conservation efforts to protect aquatic ecosystems.

## Introduction

In recent decades, the release of pharmaceutical chemicals into aquatic habitats has grown to be a significant ecological problem (Mezzelani et al., 2018). Pharmaceutical pollutants originate from various sources, including human and veterinary medicine, and their continuous release into water bodies raises concerns about their potential risks to aquatic ecosystems (Chaturvedi et al., 2021). These pollutants, which comprise a broad range of pharmaceuticals, find their way into water bodies through a variety of channels, including agricultural runoff, sewage effluents, and inappropriate disposal of leftover medication (Khan et al., 2020). Due to their persistent nature and limited degradation, many pharmaceuticals can accumulate in the environment, leading to long-term exposure in aquatic organisms (Yang et al., 2021). Because of their strong biological action, these medications can be extremely dangerous to aquatic species even at low environmental concentrations (Felis et al., 2020). Unlike other organic pollutants, pharmaceuticals are specifically designed to interact with biological systems, which increases the likelihood of unintended toxic effects on non-target organisms (Suman et al., 2022). Pharmaceuticals are intended to produce particular therapeutic benefits at low dosages; however, they can unintentionally influence non-target species, which can disrupt the ecosystem (Caldwell et al., 2014; Kock et al., 2023).

Several classes of pharmaceuticals have been detected in aquatic environments, including antibiotics, anti-inflammatory drugs, beta-blockers, and anxiolytics (Corcoran et al., 2010; Khetan and Collins, 2007). Among the many medications found in aquatic environments, sotalol, naproxen, bromazepam, and metoprolol have received special attention. Bromazepam is widely used for its anxiolytic properties (Vieira et al., 2022), naproxen is commonly used as non-steroidal anti-inflammatory drugs, NSAID, (Day and Graham, 2013), and metoprolol and sotalol are beta-blockers employed in the treatment of cardiovascular conditions (Kühlkamp et al., 2002; Martínez-Milla et al., 2019). These compounds have been detected in surface waters, wastewater treatment plant effluents, and even drinking water sources at concentrations that may pose significant ecological risks (Koné et al., 2013; Sanusi et al., 2023). They are constantly present in water bodies because to their widespread use, which can have a variety of biological consequences on aquatic life (Ali et al., 2019; Bottoni et al., 2010; Pal et al., 2010).

The ecological impacts of pharmaceuticals on aquatic life are multifaceted, encompassing various physiological and biochemical parameters (Porretti et al., 2022). Neurological, immunological, and histopathological changes are three critical indicators of the adverse effects of pharmaceutical contaminants. Neurological parameters provide insight into the effects of pharmaceuticals on the nervous system of aquatic organisms, potentially leading to altered behavior and impaired sensory functions (Vaudin et al., 2022). The nervous system and neurological response mechanism of aquatic creatures, appear to be sensitive to a variety of pollutants, including pesticides, heavy metals, and some medications (Rhee et al., 2013). The presence of pharmaceutical pollutants in aquatic ecosystem leads to dramatically reduced the amount of acetylcholinesterase (AChE) activity in the brain, and this inhibition was correlated with the cumulative quantity of the pollutants (Liu et al., 2017). AChE activity was dramatically reduced in *Carassius auratus* fish treated to sulfamethoxazole (Yang et al., 2019). Acetylcholine builds up in the neurotransmitter when AChE activity is inhibited. Furthermore, behavioral states are correlated with brain neurotransmitter levels and enzyme function (Scott and Sloman, 2004). Therefore, other major physiological functions like development, food consumption, energy metabolism, and considerable mortality may be affected by these possible changes in the physiological activities (Rhee et al., 2013). Immunological parameters can reveal how contaminants impact the immune system, affecting the organism’s ability to fend off pathogens and stress. Previous study states that frequent exposure to chemical pollutants as nonylphenol or physical agents as UVA may have detrimental effects on the immune and physiological systems of fish, which could pose major ecological risks to the viability of fish populations in their natural habitat (Ahmadpanah et al., 2019; Sayed et al., 2016; Soliman et al., 2019). While some fish reactions varied depending on the drug contaminant, previous findings indicates that exposure to diclofenac and dexamethasone at trophic levels can cause immunotoxic effects and hematological alterations that negatively affect aquatic fish (Ribas et al., 2016).

Common carp (*Cyprinus carpio*) is a widely studied species in ecotoxicology due to its ecological and economic importance, as well as its sensitivity to environmental changes (Yancheva et al., 2019). It serves as an excellent bioindicator species for assessing the impact of pollutants, including pharmaceutical contaminants, in aquatic environments (Islas-Flores et al., 2014). The neurological, immunological, and histopathological responses of common carp to pharmaceutical exposure can provide valuable data for understanding the broader ecological effects of these contaminants.

For instance, exposure to bromazepam in aquatic environments can lead to neurological alterations such as reduced activity levels and impaired stress responses, as well as histopathological changes such as gill and liver damage in fish (Carvan and Bradbury, 2008; Gonzalez-Rey, 2012; Olivares-Rubio and Arce, 2023). Similarly, naproxen has been shown to cause histological alterations in the liver and kidney tissues of exposed fish and can affect immunological parameters, demonstrating the compound’s toxicological impact (Świacka et al., 2021). Fish exposed to metoprolol and sotalol have been linked to histopathological indicators, developmental abnormalities, and altered neurological function (Dediu et al., 2022; Marin-Morales et al., 2016).

Understanding the environmental fate and impact of these pharmaceuticals is crucial for assessing their ecological risk. This study aims to evaluate the neurological, immunological, and histopathological responses of common carp (*C. carpio*) to bromazepam, naproxen, metoprolol, and sotalol. By investigating these specific parameters, the study will contribute to the broader understanding of pharmaceutical contaminants’ ecological effects, informing risk assessments and guiding environmental protection strategies.

## Materials and methods

The animal use and care committee of Assiut University in faculty of science, Egypt, accepted the established protocol (Protocol No., 07\2024\0056) of this study.

### Chemicals

Bromazepam (CAS Number:1812-30-2), Naproxen CAS No.:22204-53-1), metoprolol (CAS No.:56392-17-7), and sotalol (CAS No.:959-24-0) were purchased from Sigma Aldrich (Deisenhofen, Germany).

### Sampling and acclimatization of the experimented crayfish

Fishermen used the proper mesh size to catch *C. carpio* from the Nile in the Assiut governorate, Egypt, and kept the fish in cages in their natural habitat. Common carp were brought in aerated Nile water to the Fish Biology and Pollution lab at the Faculty of Science, Assiut University, Egypt, once a sufficient number had been gathered. After that, the fish were given the suggested circumstances for 2 weeks to acclimate. Dechlorinated water with a pH of 7–8 and more than 6 mg/L of dissolved oxygen was used as experimental water. The experiment was conducted with oxygen supplied constantly. The water temperature was maintained at 25°C ± 2°C, with a total hardness of 150 ± 10 mg/L as CaCO_3_ and an ammonia concentration below 0.02 mg/L.

The fish were fed a standard commercial diet once daily, and uneaten food was removed to maintain water quality. Water parameters were checked daily to ensure stability. Throughout the experiment, fish health was monitored daily for signs of disease, stress (e.g., erratic swimming, loss of equilibrium), and abnormal behavior. However, no mortality or noticeable behavioral changes were observed in any of the experimental groups, including the control group. Five triplicate groups of common carp (40.5 ± 6.74 gm) were used (30 fish/group). The first group is the control group without any treatment. The second group was treated with bromazepam (15.54 μg/L), third group treated with naproxen (14.40 μg/L), fourth group treated with metoprolol (7.76 μg/L), and the fifth group treated with sotalol (3.33 μg/L). The pharmaceuticals were dissolved directly in experimental water to simulate waterborne exposure, mimicking real environmental contamination scenarios. After 15-day exposure, six fish were chosen for the proceeding analysis in accordance with the required inquiry.

### Assessment of neurological markers

Brain samples of six fish/group, were homogenized using 10 L of cold Tris buffered saline as described by (Hamed et al., 2022). The homogenate was centrifuged for 10 min at 3,000 rpm and 4°C. Then, using commercial kits (Nanjing Jiancheng Bioengineering Institute, Nanjing, China), the quantities of acetylcholinesterase (AChE), MAO, and nitric oxide (NO) in the supernatant were determined. Protein concentrations were measured using a Bradford protein assay (Bradford, 1976) to standardize activity, using bovine serum albumin as the reference. AChE activity in brain homogenates was assessed using a kit and standard procedures (Ellman et al., 1961). By calculating the quantity of hydrolyzed micromoles of acetylthiocholine iodide per minute per microgram of protein, the activity of 1 U of AChE was ascertained. AChE activity was expressed in units per milligram of protein (U/mg protein) for the brain homogenates. The process by which MAO reacts with the substrate aniline hydrochloride to produce benzyl aldehyde was determined using recognized procedures in order to estimate MAO activity (Tabakoff and Alivisatos, 1972). The amount that raised the absorbance by 0.01 at 37°C was considered to be one unit (U) of MAO activity, which is expressed as U/mg protein. In brain homogenates, the NO concentration was expressed as micromoles per milligram of hippocampal protein (μmol/mg protein) according to Stuehr et al. (1989).

### Assessment of immune parameters

Using centrifugation at 5,000 rpm for 10 min, serum was collected from the blood samples of six fish per group (two per replication). The serum samples were then kept at −80°C until the examination of the immunological biomarkers was completed. Using the turbidity assay method, the amount of lysozyme (LYZ) activity (mg/mL) was determined (Parry Jr et al., 1965), in compliance with a previously published methodology (Overkamp et al., 1988). Interleukin-β and interleukin-6 (IL-1β and IL-6) levels were measured using an ELISA kit (CSB-E13259 Fh) and a CSB-E13258 Fh, respectively, according to Wang et al. (2009), Hanington and Belosevic (2007).

### Histological examination

Six fish from each group had their liver and kidney tissues sampled for histopathology. These samples were subsequently preserved in neutral buffered formalin and processed by a conventional automated technique (clearing with methyl benzoate and dehydrating with a graded ethanol concentration), embedded in paraffin wax, and sectioned at a five micron thickness (Biancotti et al., 2020). Hematoxylin and eosin (H&E) staining is administered after the slides have been de-waxed and rehydrated (Feldman and Wolfe, 2014). Finally, an Olympus CH30 microscope was employed for both photography and analysis.

### Statistical analysis

The statistical analysis was conducted using GraphPad Prism 9.0 (GraphPad Software, Inc.), and all data were presented as mean ± standard error of the mean (SEM). Prior to conducting ANOVA, data normality was assessed using the Shapiro-Wilk test, and homogeneity of variance was checked using Levene’s test. One-way ANOVA was conducted for comparisons among groups, followed by Fisher’s LSD test for *post hoc* analysis when assumptions were met. The threshold for statistical significance of differences between treatments and controls was set at P < 0.05. An asterisk superscript (*) was used to visually denote the significant level (p < 0.05, **p < 0.01, ***p < 0.001, ****p < 0.0001). Additionally, a *post hoc* power analysis was conducted to ensure that the sample size was sufficient to detect significant effects.

## Results

### Neurological parameters

**FIGURE 1:**
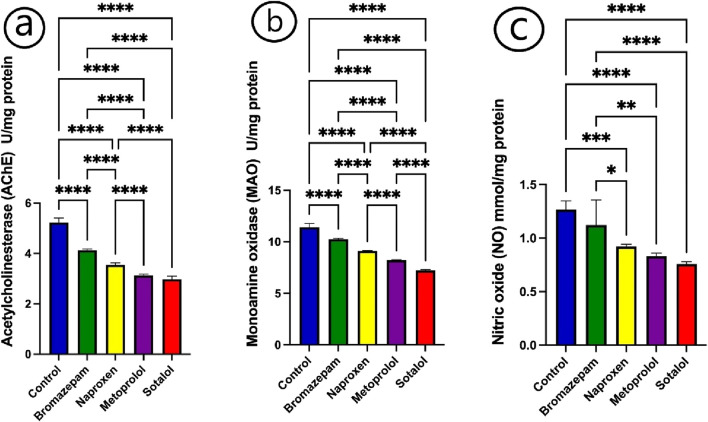
Impact of bromazepam, naproxen, metoprolol, and sotalol on common carp neurological parameters. **(a)** Acetylcholinesterase (U/mg), **(b)** Monoamine oxidase (U/mg), and **(c)** Nitric Oxide (mmol/mg). The mean ± SE is used to present the data (n = 6). Asterisks indicate significant differences from the control group (P < 0.05, *; P < 0.01, **; P < 0.001, ***; and P < 0.0001, ****).

After being exposed to bromazepam, naproxen, metoprolol, and sotalol for 15 days, the enzyme activity of acetylcholinesterase (AChE), monoaminoxidase (MAO), and the quantity of nitric oxide (NO) were considerably lowered (P < 0.05) in comparison to the control group (Figures 1a–c). The lowest value for the three enzymes was recorded for fish exposed to sotalol treatment. The AChE and MAO activities were changed significantly (p ≤ 0.05) in all treated groups compared to each other. While NO was non-significantly changed between the fish treated with naproxen, metoprolol, and sotalol groups (Figure 1c).

### Immunological parameters

**FIGURE 2:**
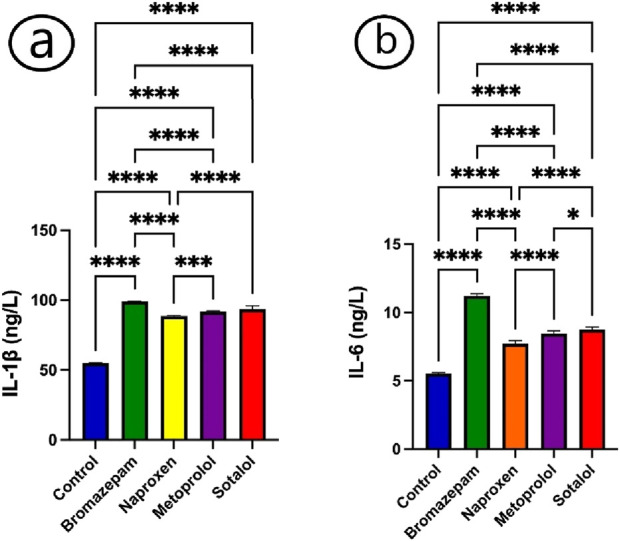
Impact of bromazepam, naproxen, metoprolol, and sotalol on common carp immunological parameters. **(a)** IL-1β(ng/L) and **(b)** IL-6 (ng/L). The mean ± SE is used to present the data (n = 6). Asterisks indicate significant differences from the control group (P < 0.05, *; P < 0.01, **; P < 0.001, ***; and P < 0.0001, ****).

Following exposure to bromazepam, naproxen, metoprolol, and sotalol, theserum of common carp showed a considerable rise in the cytokines, IL-1β and IL-6 (Figures 2a, b). There was a significant increase in IL-β and IL-6 in all treated groups relative to the control group (Figures 2a, b).

### Histopathological analysis

**FIGURE 3:**
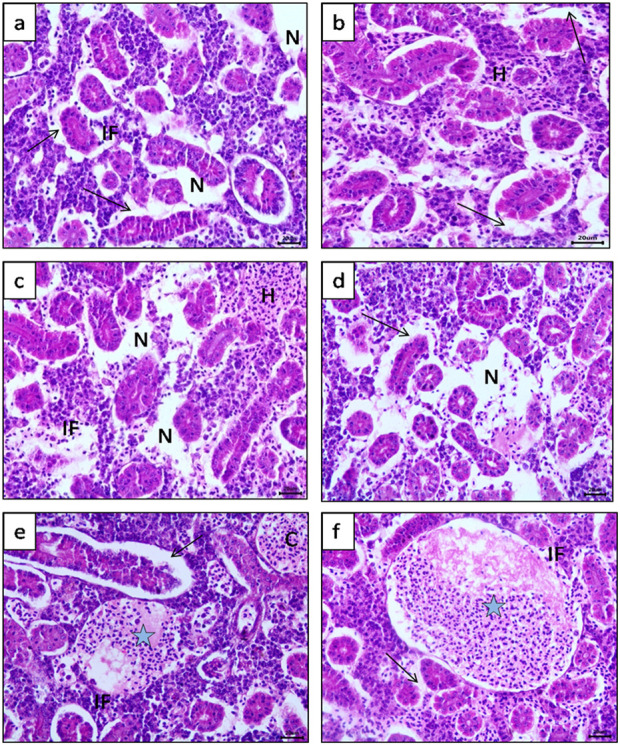
Photomicrograph of common carp (*Cyprinus carpio*) kidney (H&E) stained, showing, **(a, b)** Separation of tubular epithelium from its basement membrane (Arrow) with interstitial inflammatory cell infiltration (IF) and focal hemobiotic tissue necrosis (N). **(c, d)** Multi focal area of hemobiotic tissue necrosis (N) with diffuse Interstitial hemorrhages (H) and inflammatory cell infiltration (IF) between renal tubules were also observed. **(e, f)** Enormous thrombus become visible (Blue star) inside blood vessels with clear separation of tubular epithelium from basement membrane (Arrow) also dilatation and congestion of blood vessels (C).

**FIGURE 4:**
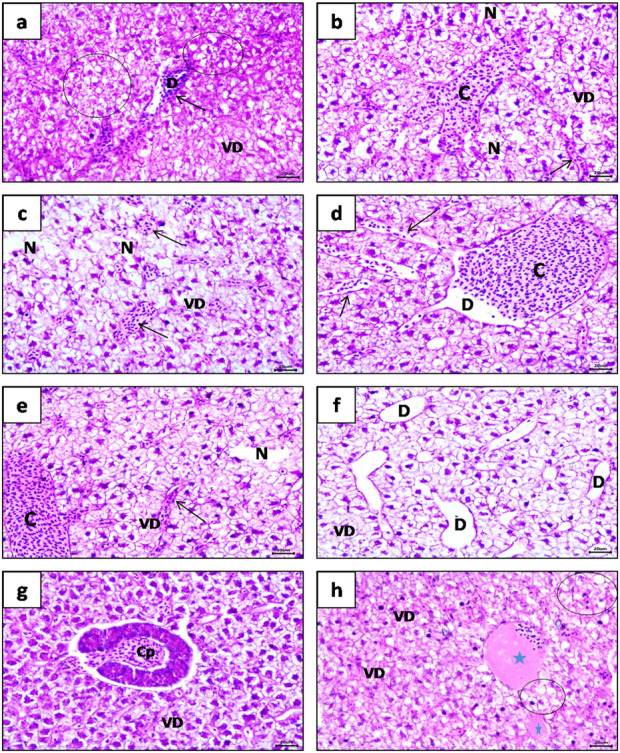
Photomicrograph of common carp (*Cyprinus carpio*) liver (H&E) staining, showing, **(a)** Massive vacuolar degeneration (VD) and fatty degeneration (Circle) in focal areas accompanied with dilatation (D) and congestion of sinusoids (arrow). **(b, c)** Multi focal area of hepatic necrosis (N) and vacuolar degeneration (VD) with mild dilatation and congestion of central veins (C) and sinusoids (Arrow). **(d, e)** Focal area of necrosis (N) with sever dilatation and congestion of central veins (C) and sinusoids (Arrow) were observed obviously. **(f)** Significant dilatation of sinusoids (D) with focal vacuolar degeneration of hepatocytes (VD). **(g)** Apparent congestion of hepatopancreas (CP). **(h)** Organized thrombus of blood vessels (Blue star) with focal vacuolar degeneration (VD) and fatty degeneration (steatosis) (Circle).

Figures 3, 4 show the histopathological effects of four commonly used pharmaceuticals (bromazepam, naproxen, metoprolol, and sotalol) on the kidneys and liver of common carp (*C. carpio*). The results highlight significant tissue damage, with some drugs causing more severe effects than others.

Kidney Histopathology, compared to the control group, all treated fish exhibited various degrees of kidney damage. However, bromazepam and naproxen had the most severe impact. Key pathological changes observed included diffuse necrosis of renal tubular epithelium, narrowing of tubular lumens, and separation of the epithelium from the basement membrane, leading to extensive inflammatory cell infiltration (Figures 3a, b). Additionally, multi-focal areas of hemobiotic necrosis were prominent, along with focal perivascular inflammatory reactions (Figures 3c, d). The Bowman’s space exhibited mild dilatation with glomerular tuft atrophy, while vascular congestion and degeneration of blood vessel walls were clearly evident (Figure 3e). Severe hemorrhages and thrombus formation were particularly noticeable in the bromazepam-treated group (Figure 3f), suggesting significant renal damage.

Liver Histopathology, Liver sections from the treated fish showed substantial structural deterioration compared to the control group, with naproxen and metoprolol causing the most severe damage. The major findings included severe hepatocellular degeneration, vacuolar degeneration, and fatty degeneration (steatosis), along with marked vascular dilatation (Figure 4a). The presence of multi-focal hepatic necrosis was one of the most diagnostic features, appearing clearly in treated groups (Figures 4b, c). Additionally, central vein dilatation, congestion, and sinusoidal expansion were particularly prominent in the naproxen-treated group (Figures 4d–f). Hepatopancreatic congestion was observed across all treated groups (Figure 4g), while organized thrombus formation was most pronounced in the metoprolol and naproxen groups (Figure 4h).

## Discussion

### Neurological parameters

The study examines the effects of various pharmaceuticals as bromazepam, naproxen, metoprolol, and sotalolon acetylcholinesterase (AChE), monoamine oxidase (MAO), and the levels of nitric oxide (NO) in common carpafter 15 days of exposure. A significant reduction in AChE and MAO activities (P < 0.05) was observed across all treated groups, suggesting that these pharmaceuticals interfere with essential neurological processes. By regulating acetylcholine’s activity, AChE is essential for synaptic transmission at cholinergic synapses (Basha et al., 2012). Moreover, MAO is essential for the metabolism of neurotransmitters such serotonin, dopamine, adrenaline, and norepinephrine (Devi et al., 2005). Numerous mental illnesses have been linked to AChE and MAO dysfunctions (Zou et al., 2013). We concluded that the changed affinity for free-SH groups and subsequent inhibition of their activity may be the cause of the suppression of AChE and MAO activities under TBT stress, which is consistent with the results of earlier investigations (Li et al., 2010). Nitric oxide was also significantly reduced in the treated fish compared to the control group (P < 0.05), highlighting an overall negative impact on the nitric oxide signaling pathways. There were significant differences in the activities of these enzymes among all treated groups (p ≤ 0.05). This implies that each drug has a unique impact on these enzymes, reflecting their different modes of action or metabolic pathways. While NO levels were significantly reduced in all treated groups compared to the control, the changes in NO levels among fish treated with naproxen, metoprolol, and sotalol were not statistically significant when compared to each other. It may be possible that pharmacokinetic differences between the drugs influenced nitric acid production to varying extents. Factors such as drug metabolism, mode of action, and interaction with nitric oxide synthase (NOS) pathways may play a role in these variations (Serafim et al., 2012). Additionally, while our sample size was sufficient for detecting major effects, subtle changes may require larger sample sizes to achieve statistical significance. This suggests a relatively similar impact of these three drugs on NO production. NO is crucial for cell signaling, neurotransmission, cell protection, and regulatory actions in a variety of cells (Nava-Ruíz et al., 2010). Studies on Atlantic salmon have been conducted on this species (Holmqvist and Ekström, 1997). Consequently, changes in NO production could be a contributing cause to the emergence of neurotoxicity (Kim et al., 2011). Nitric oxide plays a crucial role in mitochondrial energetics, oxidative stress regulation, and immune responses. Previous studies have demonstrated that NO influences mitochondrial function by modulating cytochrome c oxidase activity and reactive oxygen species (ROS) production, which in turn affects metabolic efficiency and stress responses in fish (Gayathry et al., 2018). Considering these findings, we have elaborated on how the observed decrease in NO levels in our study may contribute to impaired neurological and metabolic functions, potentially influencing fish behavior and overall physiological homeostasis. Bromazepam, an anxiolytic, reaches higher concentrations in brackish and marine systems than in freshwater environments, where neuroactive pharmaceuticals are known to affect fish behavior, growth, and condition (Duarte et al., 2022). Chronic exposure to bromazepam may have sedative effects on fish, with potential sex-dependent behavioral impacts. Similar to other benzodiazepines like diazepam, bromazepam could disturb behavioral traits during courtship, potentially affecting reproduction. Additionally, it may alter neurotransmitter levels, such as AChE, in the fish brain (Chen et al., 2021). Chronic exposure to naproxen has been shown to impact fish survival and reproduction, with potential endocrine-disrupting effects (Kwak et al., 2018). Studies indicate that exposure to naproxen during early life stages of fish can alter gene transcription related to steroidogenic pathways, leading to imbalances in sex hormones. While the concentration required to produce these effects is higher than those typically found in ambient water, long-term exposure to environmentally relevant levels may still pose risks to aquatic organisms, warranting further investigation.

### Immunological parameters

The study investigated the effects of bromazepam, naproxen, metoprolol, and sotalol on the immune response of common carp, focusing specifically on the levels of the cytokines IL-1β and IL-6 in their serum. Exposure to each of the four drugs, bromazepam, naproxen, metoprolol, and sotalol, resulted in a significant increase in the levels of the cytokines IL-1β and IL-6 in the serum of common carp compared to the control group. This suggests that all the tested drugs induce an inflammatory response in the fish. The increase in IL-1β and IL-6 levels was significant in all treated groups, indicating a robust inflammatory response triggered by these pharmaceuticals (Zhang et al., 2019). The results highlight that exposure to these common pharmaceuticals can elicit a significant inflammatory response in common carp, as evidenced by elevated levels of the cytokines IL-1β and IL-6. IL-1β and IL-6 are key pro-inflammatory cytokines involved in the regulation of immune responses (Wang et al., 2022). Their elevated levels indicate that the fish are experiencing an inflammatory reaction, which could be a defense mechanism against the perceived chemical stress or toxicity caused by these drugs (Lushchak, 2016). The increase in these cytokines suggests that the immune system of the fish is being activated (Reyes-Cerpa et al., 2012). While this might be beneficial in short-term defense, chronic inflammation can lead to tissue damage and impaired physiological functions (Xu et al., 2021). The observed upregulation of IL-1β and IL-6 in response to pharmaceuticals could indicate an adaptive response aimed at neutralizing cellular stress and maintaining homeostasis. However, prolonged or excessive cytokine production may reflect a maladaptive inflammatory reaction, leading to immune dysregulation, oxidative stress, and potential tissue damage. Similar immune disruptions have been documented in studies on immunosuppressive pharmaceuticals, which affects both vertebrate and invertebrate immunity (Netea et al., 2016). These findings highlight potential ecological risks, as long-term exposure to pharmaceutical contaminants in aquatic environments could impair fish immune responses, reducing resilience to infections and environmental stressors (Corcoran et al., 2010; Kataoka and Kashiwada, 2021; Limbu et al., 2018; Overturf et al., 2015; Świacka et al., 2021).

### Histopathological analysis

Histopathology, used as warning of various pollutants effects on overall health of the entire ecosystem inhabitants and also considered as a vital technique in aquatic toxicity (Khoshnood et al., 2010), histopathological changes in fish represents a practical tool to review the degree of Water pollution. Fish histopathological alterations serve as a useful technique for assessing the level of water contamination. One important class of xenobiotics found in the environment are pharmaceuticals. Many of these substances are essential to human life, and in recent decades, their use has rapidly expanded for both livestock husbandry and the treatment of human illnesses (Bernhardt et al., 2017). Similarly, drugs may have significant sub-lethal ecological effects on aquatic species (Brodin et al., 2014). Because of its extensive distribution and ease of use in the lab, the common carpa fish with significant economic valuewas chosen for the bioassay studies (Aydın and Köprücü, 2005). According to Bawuro et al. (2018) the liver is the primary organ responsible for the detoxification, conversion, and storage of harmful substances in fish. Like in higher vertebrates, the kidney plays a crucial role in maintaining a stable internal environment, electrolyte balance, and water balance in fish. Since the kidney of fishes gathers the majority of post-branchial blood, renal abnormalities may be considered reliable markers of environmental contamination (Cengiz, 2006).

Our present histopathological results showed that after exposing common carp (*C. carpio*) for 15 days to bromazepam, naproxen, metoprolol, and sotalol showed clear adverse similar liver pathological alterations revealed, distinct deterioration of hepatic architecture, vacuolar degeneration accompanied with fatty degeneration in parts of hepatic tissue (steatosis), Multi focal area of hepatic necrosis, vascular dilatation and congestion, dilatation and congestion of hepatopancrease and thrombosisNonetheless, the findings revealed some variance in the level of manifestation of every histological change in the fish liver across the four widely used medications.

In accordance with earlier research, Hoseini et al. (2022) documented the combined effects copper and/or microparticles on the liver of *C. carpio* over a 14-day period. Exposure to copper and/or PVC microparticles caused leukocyte infiltration, hypermeia, and an increase in the number and size of melanomacrophage centers. Such pathological alterations have been documented in MPs (Jabeen et al., 2018; Santana et al., 2022)or fish exposed to copper (Mela et al., 2013; Paulino et al., 2014; Yancheva et al., 2014). Additionally, it is frequently linked to the hepatocyte’s reaction to toxins (Ghelichpour et al., 2017; Macêdo et al., 2020).

Evidence of liver damage, which is closely related to our findings, was documented in freshwater catfish exposed to an organophosphate herbicide by Persis and Kalaiarasi (2001). Hepatocyte bleeding and disintegration, sinusoidal blood congestion, hypertrophy, cord disorganization, and lymphocyte infiltration were all examples of this injury.

The increased leukocyte infiltration in the exposed fish’s liver can be explained by inflammation, which attracts leukocytes to the injured tissue. Additionally, the appearance of hyperemia in the exposed fish can be explained by an increase in blood flow inside the inflammatory tissues. The increased volume of blood in the capillary causes congestion, which is a disruption in blood circulation (Rejeki et al., 2008). Hepatic necrosis and cellular degeneration may be caused by blood congestion (Mohamed, 2008). Liver hyperemia may unavoidably lead to hepatic necrosis and atrophy, claim (Van Dyk et al., 2007). We further hypothesize that hepatocyte necrobiosis, a manifestation of stenosis of the blood arteries, may cause changes in fish metabolism. Necrobiosis might be regarded as a warning sign of changes in fish metabolic activity, according to (Figueiredo-Fernandes et al., 2007).

In addition, Georgieva et al. (2016) observed the hepatic morphological structure of common carp in response to the water quality in an area with heavy copper mining that has been persistently metal-contaminated during the past few seasons spring, summer, and autumn 2013and found severe hepatic histological alterations, comprising necrosis, karyopyknosis, karyorrexis, karyolysis, and necrotic changes (necrobiosis); and blood vessel alterations, such as hyperemia in the major blood arteries and sinusoids.

Our findings about the histological alterations in the liver of common carp are comparable to those of Laurén et al. (1990), Monteiro et al. (2005), Paris-Palacios et al. (2000), Rajeshkumar and Munuswamy (2011), Van Dyk et al. (2007).

Nayak et al. (1996) demonstrated that rather than being cells undergoing hydropic degeneration, many of the vacuolated hepatocytes observed in liver injury are cells that have undergone adaptive modification to withstand increased exposure. It is believed that biochemical interference, including microtubule disaggregation, altered protein synthesis, decreased ion regulation, depletion of energy resources, and inhibition or activation of enzyme activity, may be the cause of the degenerative histological changes as granular, vacuolar, hydropic, and fatty degeneration (Laurén et al., 1990). Our findings were in line with those of (Varanka et al., 2001), who hypothesize that anomalies in hepatocytes and hepatic structure caused by significant metal accumulation in the liver can ultimately result in cell death. Mela et al. (2007) state that one of the most significant alterations in liver morphology caused by metals is the existence of necrotic regions. Our assessment concurred with the authors’ hypothesis that necrosis is most likely caused by compromised cell membrane integrity and disruption of fish’s glucose metabolism.

Our Histopathological observation on the kidney tissues of common carp exposed to bromazepam, naproxen, metoprolol, and sotalol for 15 days were characterized by Diffuse necrosis of renal tubular epithelium, narrowing of its lumen and significant separation from its basement membrane, focal perivascular inflammatory cell infiltrations, Multi focal area of hemobiotic tissue necrosis, dilatation of bowman’s space with atrophy of glomerular tuft,Dilatation and congestion of blood vessels with degeneration of its wall and hemorrhages. Also thrombus was clearly observable.

Histopathological changes were examined in common carp exposed to different phosalone concentrations *in vivo* under a semistatic regime for 14 days, showing a similar pattern to our findings (Kaya et al., 2013). Hepatocyte changes included nuclear degeneration, enlargement, congestion, and cytoplasmic vacuolation. Kidney tissue showed signs of glomerular capillary dilatation, mononuclear cell infiltration, tubule degeneration, and hypertrophy. According to the findings, phosalone exposure had a negative impact on the fish’s health because it caused oxidative stress.

However, under a light microscope, the histopathological changes in the gills, liver, and kidney of common carpafter they were exposed to sub-lethal levels of chlorpyrifos for 14 days were examined (Pal et al., 2012). He suggested that hepatocyte alterations be more noticeable, along with abnormal cell shape, cytoplasmic vacuolation, cytoplasmic and nuclear degeneration, cellular rupture, pyknotic nucleus, necrosis, gliding melanomacrophages, nuclear and cellular hypertrophy, cellular atrophy, and nucleus. Additionally, the kidney’s histopathological lesions were determined to be cellular and nuclear hypertrophy, tubular lumen narrowing, glomerular capillary dilatation, glomerulus degeneration, cytoplasmic vacuolation, hyaline droplet degeneration, nuclear degeneration, tubular lumen blockage, tubular regeneration, and Bowman’s space hemorrhage. These findings are consistent with our findings. According to Zou et al. (2022), oxidative stress brought on by an excess of reactive oxygen species (ROS) and antioxidant system suppression is one of the main causes of kidney damage (Demir, 2019; Gur et al., 2022). Globulomegaly (increasing glomerular size) is caused by Bowman’s space dilatation and expansion combined with glomerular tuft atrophy. Furthermore, blood vessel dilatation and congestion can raise angiotensin II levels, which in turn can raise blood pressure (Mariana et al., 2016). Furthermore, glomerular and blood vessel walls are subjected to mechanical stress from elevated blood pressure, which can cause damage, wall degeneration, and bleeding (Ichimura et al., 2013). According to Van Dyk et al. (2007), hepatic necrosis and atrophy are undoubtedly caused by degeneration and hemorrhages. The common carp’s kidney tissues showed histopathological effects after being exposed to 0.029 mg L^−1^ (50% of 96-h LC50) and 0.041 mg L^−1^ (70% of 96-h LC50) solutions of deltamethrin for 96 h. These effects included renal tubule epithelial cell degeneration, pycnotic nuclei in the hematopoietic tissue, glomerular capillary and glomerulus dilatation, intracytoplasmic vacuoles in renal tubule epithelial cells with hypertrophied cells, and finally tubular lumen narrowing (Cengiz, 2006).

The kidneys of fish exposed to lindane exhibited tubular necrosis, tubular epithelial cell vacuolization, and desquamation, which is in good agreement with our findings (Ortiz et al., 2003). Similarly, dilatation of the renal tubule lumina, tubule necrosis, vacuolation of blood cells within the glomerular tuft and glomerular tuft shrinking were reported by Badroo et al. (2020). In Heteropneustes fossilis exposed to chlorpyrifos, all of the earlier cases have been documented.

Bowman’s capsule and glomerulus dimensions significantly decreased after Elsan treatment in Channa punctatus, and cytoplasmic precipitation and karyolysis caused the tubules to lose their normal form Moniruzzaman et al. (2017). Tubule dilatation and necrotic alterations marked by karyorrhexis and karyolysis at the nuclei of impacted Labeo rohita cells subjected to hexachlorocyclohexane were noted by Das and Mukherjee (2000).

## Conclusion

This study highlights the substantial ecological risks posed by pharmaceutical contaminants in aquatic environments, specifically their neurotoxic, immunotoxic, and histopathological effects on common carp (*C. carpio*). Exposure to bromazepam, naproxen, metoprolol, and sotalol resulted in significant disruptions, including reduced acetylcholinesterase (AChE) and monoamine oxidase (MAO) activities and decreased nitric oxide (NO) levels, indicating severe neurological impairment. Furthermore, the elevated levels of pro-inflammatory cytokines interleukin-1β (IL-1β) and interleukin-6 (IL-6) suggest a strong inflammatory response. Notably, this study is the first to report histopathological alterations in the kidney and liver of *C. carpio* due to exposure to these four pharmaceuticals, filling an important knowledge gap in aquatic toxicology.

Among the four tested pharmaceuticals, bromazepam and naproxen clearly caused the most severe kidney damage, while naproxen and metoprolol had the most harmful effects on the liver. These findings indicate that exposure to these drugs can lead to significant structural and functional impairments in fish organs, raising concerns about their potential environmental risks.

The findings underscore the urgent need for stricter environmental regulations and improved wastewater treatment strategies to mitigate the impact of pharmaceutical pollutants. Given the increasing environmental concentrations of these contaminants, future studies should focus on long-term exposure effects, mixture toxicity, and the underlying molecular mechanisms driving these physiological disruptions. Additionally, expanding research into other fish species and ecological levels (e.g., population and community dynamics) will provide a more comprehensive understanding of the broader implications of pharmaceutical pollution. Protecting aquatic ecosystems requires an integrated approach that combines monitoring, risk assessment, and policy intervention to safeguard biodiversity and ecosystem health.

## Data Availability

The original contributions presented in the study are included in the article/Supplementary Material, further inquiries can be directed to the corresponding author.
